# Does the optimal position of the acetabular fragment should be within the radiological normal range for all developmental dysplasia of the hip? A patient-specific finite element analysis

**DOI:** 10.1186/s13018-016-0445-3

**Published:** 2016-10-04

**Authors:** Xuyi Wang, Jianping Peng, De Li, Linlin Zhang, Hui Wang, Leisheng Jiang, Xiaodong Chen

**Affiliations:** 1 Department of Orthopaedics, The First Affiliated Hospital of Bengbu Medical College, Bengbu, Anhui China; 2 Department of Orthopaedics, Xinhua Hospital, Affiliated to Shanghai Jiaotong University School of Medicine, Shanghai, China; 3 Department of Biomedical Engineering, Shanghai University of Technology, Shanghai, China

**Keywords:** Developmental dysplasia of the hip, Finite element analysis, Periacetabular osteotomy

## Abstract

**Background:**

The success of Bernese periacetabular osteotomy depends significantly on how extent the acetabular fragment can be corrected to its optimal position. This study was undertaken to investigate whether correcting the acetabular fragment into the so-called radiological “normal” range is the best choice for all developmental dysplasia of the hip with different severities of dysplasia from the biomechanical view? If not, is there any correlation between the biomechanically optimal position of the acetabular fragment and the severity of dysplasia?

**Methods:**

Four finite element models with different severities of dysplasia were developed. The virtual periacetabular osteotomy was performed with the acetabular fragment rotated anterolaterally to incremental center-edge angles; then, the contact area and pressure and von Mises stress in the cartilage were calculated at different correction angles.

**Results:**

The optimal position of the acetabular fragment for patients 1, 2, and 3 was when the acetabular fragment rotated 17° laterally (with the lateral center-edge angle of 36° and anterior center-edge angle of 58°; both were slightly larger than the “normal” range), 25° laterally following further 5° anterior rotation (with the lateral center-edge angle of 31° and anterior center-edge angle of 51°; both were within the “normal” range), and 30° laterally following further 10° anterior rotation (with the lateral center-edge angle of 25° and anterior center-edge angle of 40°; both were less than the “normal” range), respectively.

**Conclusions:**

The optimal corrective position of the acetabular fragment is severity dependent rather than within the radiological “normal” range for developmental dysplasia of the hip. We prudently proposed that the optimal correction center-edge angle of mild, moderate, and severe developmental dysplasia of the hip is slightly larger than the “normal” range, within the “normal” range, and less than the lower limit of the “normal” range, respectively.

**Electronic supplementary material:**

The online version of this article (doi:10.1186/s13018-016-0445-3) contains supplementary material, which is available to authorized users.

## Background

Bernese periacetabular osteotomy (PAO), as developed by Ganz et al. [1, 2], has been used for the treatment of developmental dysplasia of the hip (DDH) in adolescents and adults for more than 30 years. Good to excellent prognosis has been reported by many scholars [3–5]. Throughout the postoperative follow-up, however, we found that hip pain did not improve significantly and the cartilage degeneration continued to deteriorate for some DDH patients, even though their acetabular angle had been corrected into the “normal” range. The degree of correction for the acetabular fragment is not always directly proportional to the improvement of clinical symptoms, but this biomechanical mechanism has rarely been investigated.

At present, there are two main methods of biomechanical analysis: experimental biomechanics [6–9] and theoretical biomechanics [10, 11]. Experimental biomechanics is not widely used in the clinic due to the unavailability of cadaver specimens with acetabular dysplasia and the fact that the experiment cannot be repeated. Theoretical biomechanics is based on a mathematical model for numerical analysis, such as finite element analysis (FEA), which can handle different complex geometries and can provide various non-invasive mechanical tests [12–14]. However, FEA is a time-consuming, expensive, and professional task, impossible to perform in every DDH patients preoperatively under present social and economic conditions.

We therefore asked (1) whether the correction of the acetabular fragment into the so-called radiological “normal” range is the best choice for all DDH patients with different severities of dysplasia? (2) If not, is there any correlation between the biomechanically optimal position of the acetabular fragment and the severity of dysplasia in DDH patients? (3) Can we take advantage of this correlation and determine the optimal position of the acetabular fragment by measuring the morphological parameters of DDH without the help of FEA preoperatively?

Based on these questions, we selected three DDH patients (four hips) with different severities of dysplasia, performed virtual PAO on the FE model, and calculated the contact area and pressure, as well as von Mises stress in the articular cartilage as the acetabular fragment was corrected to different positions. Our aim was to find the optimal position of the acetabular fragment for DDH with differing dysplastic severities as well as the relationship between them and to offer valued help for surgeons formulating customized surgical planning.

## Methods

All investigations were conducted in conformity with the Helsinki Declaration, and the study protocol was approved by our institutional review board (Ethics Committee of Xin Hua Hospital affiliated to Shanghai Jiao Tong University School of Medicine, Approval No. XHEC-D-2016-008).

Three dysplastic hips and one normal hip of three female patients with different severities of acetabular dysplasia were analyzed. To eliminate the effects of proximal femoral deformities on the numerical prediction, none of the patients were accompanied with hip varus or valgus, cam deformity of femoral head-neck junction. The clinical data for each patient are summarized in Table 1. A virtual PAO was performed with the acetabular fragment rotated anterolaterally to incremental center-edge (CE) angles, and the contact area and pressure and von Mises stress in the articular cartilage were calculated at different CE angles.

**Table 1:** Clinical data of the three patients

Patient (NO)	Gender	Age (years)	BMI (kg/m^2^)	LCEA (°)	ACEA (°)	Contact area (mm^2^)	Contact pressure (MPa)	von Mises stress (MPa)
1 (left)	Female	21	22	32	52	387.204	4.728	1.632
1 (right)	Female	21	22	19	47	327.067	5.759	2.169
2	Female	28	20	7	22	276.247	6.328	2.393
3	Female	29	24	−7	11	225.634	6.935	2.514

*BMI* body mass index, *LCEA* lateral center-edge angle, *ACEA* anterior center-edge angle

**Fig. 1:**
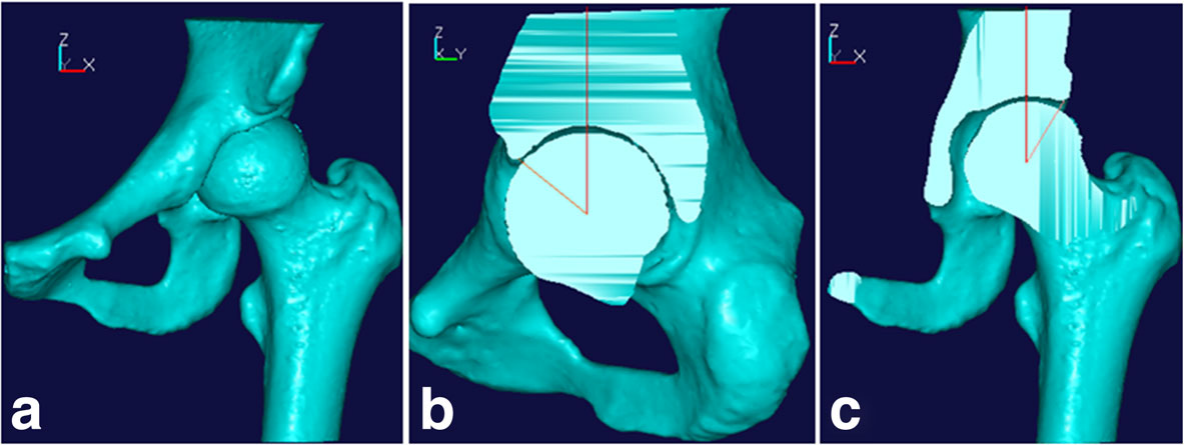
The measurement methods of acetabular angle on 3D model. **a** The 3D reconstructed model using original CT data. **b** The sagittal image passing through the center of the femoral head; the intersection angle represents ACEA. **c** The coronal image passing through the center of the femoral head; the intersection angle represents LCEA

The severity of acetabular dysplasia of each subject was determined by measuring the acetabular angle and then comparing it with the value of normal hip preoperatively. Because the reliability and accuracy of the measurement based on conventional two-dimensional (2D) images (e.g., X-ray and 2D-CT) is susceptible to the positional variables, three-dimensional computerized tomography (3D-CT) reconstruction was used in this study. The lateral center-edge angle (LCEA) and the anterior center-edge angle (ACEA) of the normal hip are approximately 32° and 54°, respectively, according to our previous research results [15], which is larger than the angles measured in 2D images (Fig. 1).

**Fig. 2:**
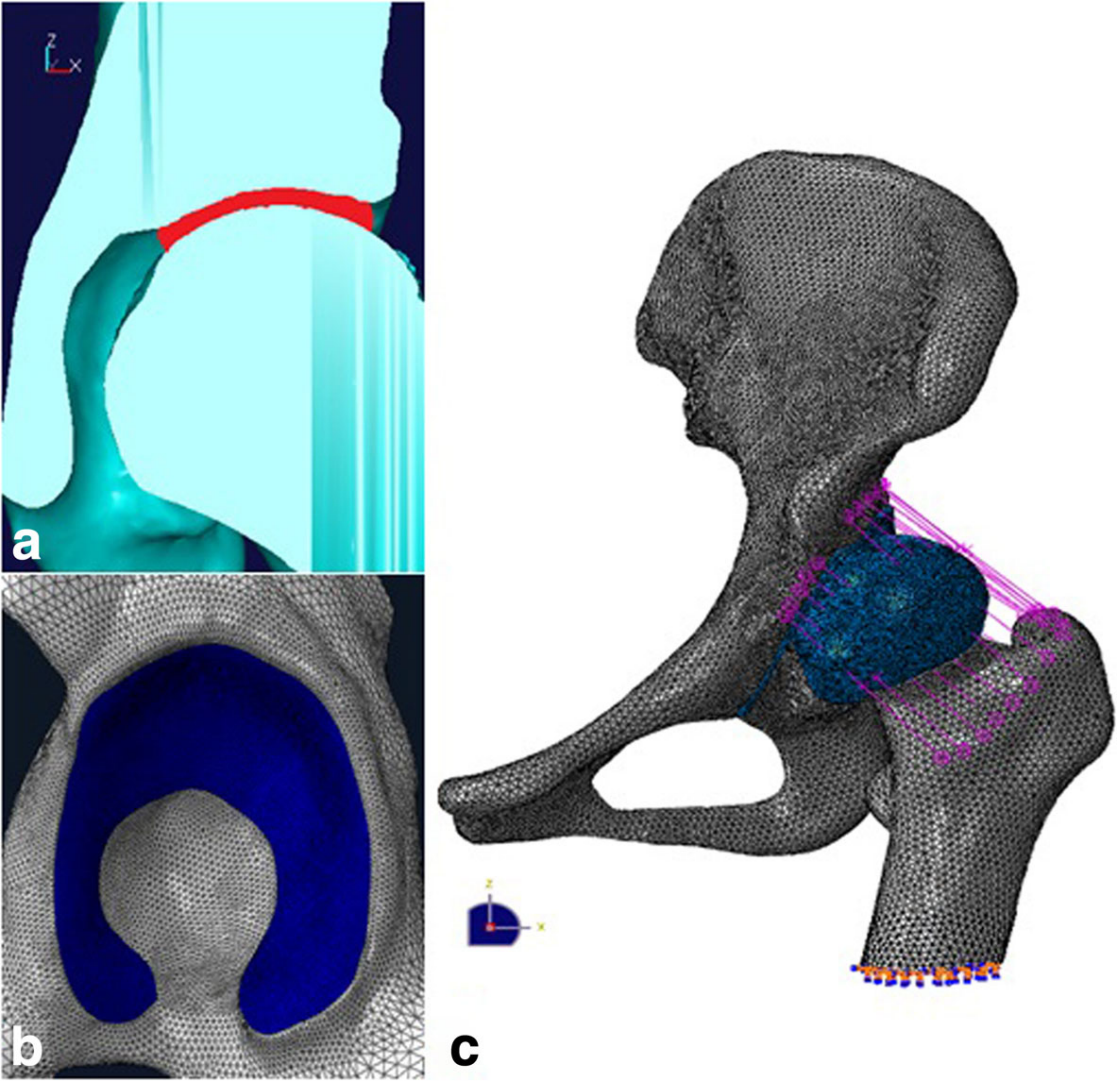
The generation of articular cartilage and 3D FE model of the hip. **a** A red filling layer was constructed in the joint space between the femoral head and acetabulum; the bisected part of this layer was taken as acetabular cartilage and femoral head cartilage. **b** The constructed acetabular cartilage. **c** The constructed 3D FE model of the femur and pelvis, the blue layer represents the articular cartilage and the purple line represents the spring elements simulating the capsular ligaments

The 3D FE models of the femur and the pelvis were generated from preoperative CT scan data using the Abaqus 6.10-1 FE analysis software (Abaqus, Inc., Dassault Systemes Simulia Corp, Providence, RI) (Additional file 1: Figure S1, Additional file 2: Figure S2). Due to the various wearing degrees of articular cartilage and individual differences, the resultant cartilage layer varied in thickness from 1.5 to 2.0 mm, corresponding to the joint clearance (Fig. 2a). Given the horseshoe shape of the acetabular cartilage, the region of the acetabular fossa was excluded (Fig. 2b). In vivo biomechanical experiments showed that the acetabular labrum and peripheral soft tissues also play important roles in the process of stress distribution in the hip joint [16, 17]. We elongated the cartilage layer of acetabulum past the bone periphery of the acetabular rim by 2 mm [18], simulating the geometry of the labrum, and developed several spring elements connecting the pelvis and the proximal femur, simulating the peripheral soft tissues (Fig. 2c).

The bone and cartilage were meshed with ten node tetrahedral elements, which have four corners and sides. The number of elements and nodes were assigned using the method described by Zou et al. [19]. Different mesh densities (e.g., coarse, fine, and very fine) were used for each model to compare the average contact pressure and contact area in the articular cartilage. The optimal mesh size was chosen according to the criteria that a change in the average contact pressure and contact area in the acetabular cartilage of <1 % between different meshes. Due to the different sizes and geometries, the optimal number of elements for each model was different. In this study, the average number of elements and nodes of cortical bone were 316,529 (244,380 to 408,547) and 92,043 (69,848 to 102,792), respectively; in the trabecular bone, the average number of elements and nodes were 270,932 (226,074 to 303,851) and 61,452 (50,171 to 71,934); and the average number of elements and nodes in the cartilage were 421,439 (354,799 to 505,251) and 108,306 (92,626 to 122,078).

The bony and articular cartilage of the model were assumed to be linear elastic and isotropic material with homogeneous properties. The biphasic and viscoelastic properties of cartilage were neglected because the loads involved in this study were of a noncyclic variation. Capsular ligaments consisted of several spring elements distributed around the hip joint along its anatomic location. The detailed parameters are listed in Table 2 [20, 21]. The interface between the cartilage and bone was assumed completely bonded, and the cartilage surface between the acetabulum and femoral head was modeled as a frictionless sliding contact [22, 23].

**Table 2:** Element types and material properties

Material	Element behavior	Young’s modulus E (MPa)	Poisson’s ratio	Stiffness of spring (N/mm)
Cortical bone	Homogeneous, linear elastic, isotropic	17,000	0.3	–
Trabecular bone	Homogeneous, linear elastic, isotropic	70	0.2	–
Articular cartilage	Homogeneous, linear elastic, isotropic	15	0.45	–
Acetabular labrum	Homogeneous, linear elastic, isotropic	15	0.45	–
Teres ligament	Spring element	–	–	68 ± 25
Ischiofemoral ligament	Spring element	–		39.6 ± 24.4
Pubofemoral ligament	Spring element	–	–	36.9 ± 24.4
Inferior iliofemoral ligament	Spring element	–	–	100.7 ± 54
Superior iliofemoral ligament	Spring element	–	–	97.8 ± 67.5

**Fig. 3:**
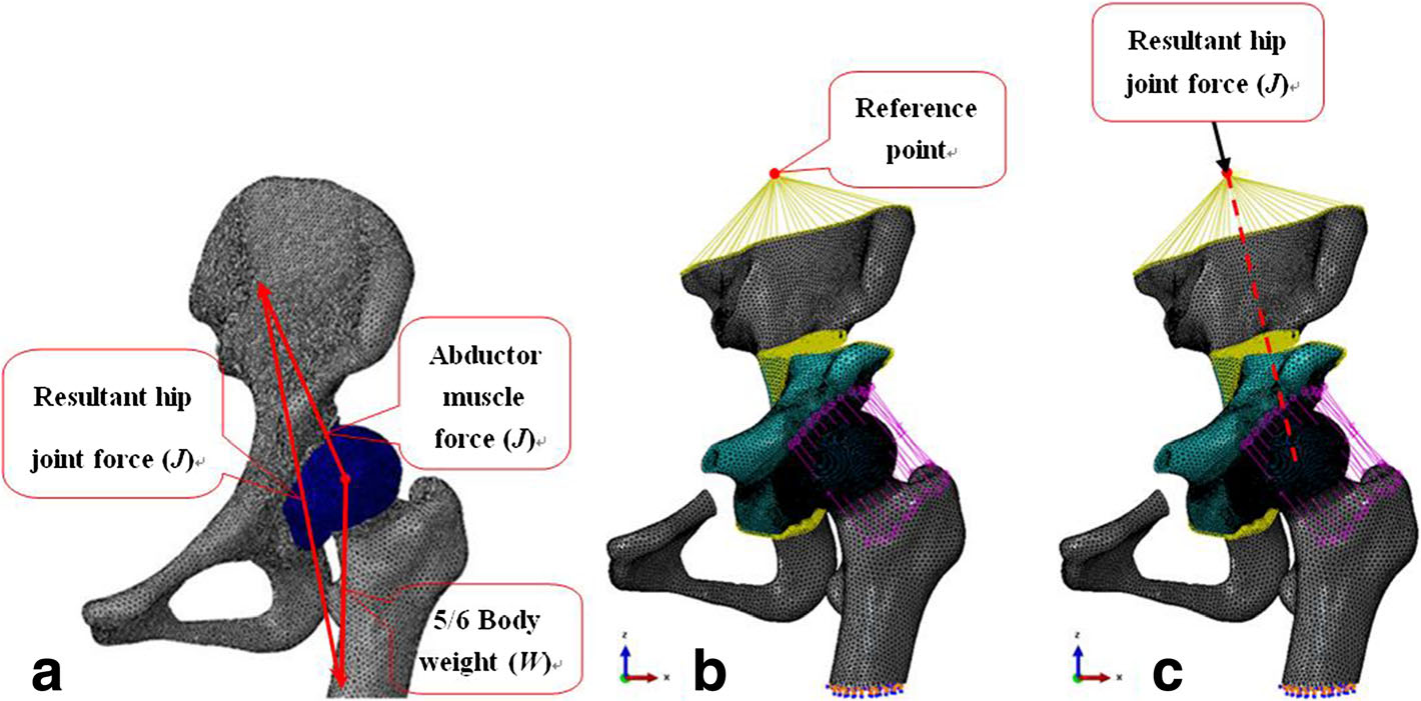
Loading and boundary conditions of FE model. **a** The stress distribution in the hip joint standing one leg (*J* resultant hip joint force; *W* 5/6 body weight; *F* abductor muscle force = 1.6 × W). **b** Set reference point and build dynamic coupling device in Abaqus; The acetabular fragment and pelvis were bounded using the “tie” constraints of Abaqus. **c** The modeled load was applied to the reference point and the line of action of the applied force passing through the center of the head

The boundary and loading conditions used in this study were based on reports from the literature resembling the status with one leg stand [19, 24]. All displacements and rotations of the distal parts of the femur were fully constrained. The resultant hip joint force represented the abductor muscle force and five sixths of the body weight standing on one foot and the line of action of the applied force passing through the center of the head (Fig. 3a). The modeled load was applied to the reference point via kinematic coupling of Abaqus (Fig. 3b). The magnitude of the applied force was patient-specific and varied with the body weight of the subject (Fig. 3c).

According to Ganz’s description [1], we performed virtual PAO on the 3D FE model. First, the acetabular fragment was rotated laterally around the center of femoral head until the LCEA closed to the “normal” range; then, the acetabular fragment was continuously rotated in 5° increments up to the “normal” range. Following every 5° of lateral rotation, the acetabular fragment was rotated anteriorly in 5° increments. The contact area and pressure and von Mises stress in the articular cartilage were calculated at each acetabular angle.

## Results

**Fig. 4:**
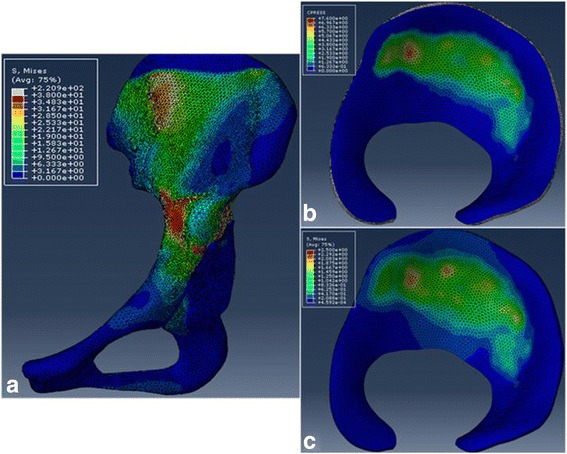
The stress distribution cloud of patient 1’s right hip. **a** The transmission of mechanics in the hip joint. **b** Contact pressure profile in acetabular cartilage. **c** von Mises stress profile in acetabular cartilage

In this study, the original acetabular angle of patient 1’s left hip exhibited an LCEA of 32° and an ACEA of 52°, which were both within the “normal” range. And the patient had no complaints of discomfort about the left hip. We therefore regarded this hip as a normally developed hip. Following the above steps, the mechanical distribution in the acetabular cartilage of patient 1’s left hip is shown in Fig. 4, the transmission of mechanics in the hip joint coincides with physiology. The contact area is wide, and the stress is dispersed in acetabular cartilage and is concentrated on the medial of the anterosuperior region, all of which are consistent with clinical observations. Furthermore, the FE model was validated indirectly by comparing the parameter predictions from this normal hip with the results obtained from cadaveric experiment [25]. In Bay’s experiments, the mean age for the cadavers was 72 years (range of 42 to 86 years). To match his experimental conditions, the Young’s modulus and Poisson’s ratio used in the validated FE model were reduced to represent the typical reduction with age. Likewise, the loading and boundary conditions applied in the model validation corresponded to the conditions employed in cadaveric experiment. The contact area and pressure of the validated FE model were 387.204 mm^2^ and 4.728 MPa, respectively, which were lower than Bay’s experimental contact area of ~418 mm^2^ and pressure of 5.35 MPa. Nevertheless, the parameter predictions were comparable to Anderson’s results, which ranged from 321.9 to 425.1 mm^2^ of contact area and 4.4 to 5.0 MPa of contact pressure [26]. Racial diversity between the subjects may be the cause of these discrepancies; the subjects selected in this study were all young Asian females who have a smaller size and a lighter weight compared with the participants in other studies.

On the whole, the trend of the parameter predictions changed to a single peak curve with the acetabular fragment had been rotated. Within a certain interval, the contact area gradually increased with the decrease of average contact pressure and von Mises stress during the rotation of acetabular fragment. However, after a critical point had been reached, the contact area did not significantly improve, and instead, the average contact pressure and von Mises stress would increase with the additional rotation of the fragment, even though the coverage of femoral head was increased (data shown in Additional file 3). This may be due to the subluxation of the hip, a mismatch between the curvatures of the acetabulum and the femoral head, which indicated that greater acetabular coverage does not necessarily mean a greater contact area between articular cartilage. Therefore, the actual contact area of the articular cartilage has a maximum critical value. When the hump of the curve was reached, the actual contact area between the articular cartilage would not increase further.

FE predictions also show that every patient has her own optimal correction of the acetabular fragment that maximizes contact area, while minimizing contact pressure and von Mises stress in the articular cartilage (Table 3). However, the optimal position of the acetabular fragment was not corrected with the acetabular angle into the so-called radiological “normal” range for all three DDH patients. Furthermore, the maximum contact area of patients 3 and 2 was lower than patient 1’s left hip (the normal hip), while their acetabular fragment was corrected to the biomechanically optimal position. The minimum von Mises stress and contact pressure of patients 3 and 2 were bigger than patient 1’s left hip (data shown in Additional file 3).

**Table 3:** Correction degree of the acetabular fragment to achieve the optimal position

	Lateral rotation (°)	Anterior rotation (°)	Final LCEA (°)	Final ACEA (°)
Patient 1 (right hip)	17	0	36	58
Patient 2	25	5	31	51
Patient 3	30	10	25	40

*LCEA* Lateral center-edge angle, *ACEA* Anterior center-edge angle

**Fig. 5:**
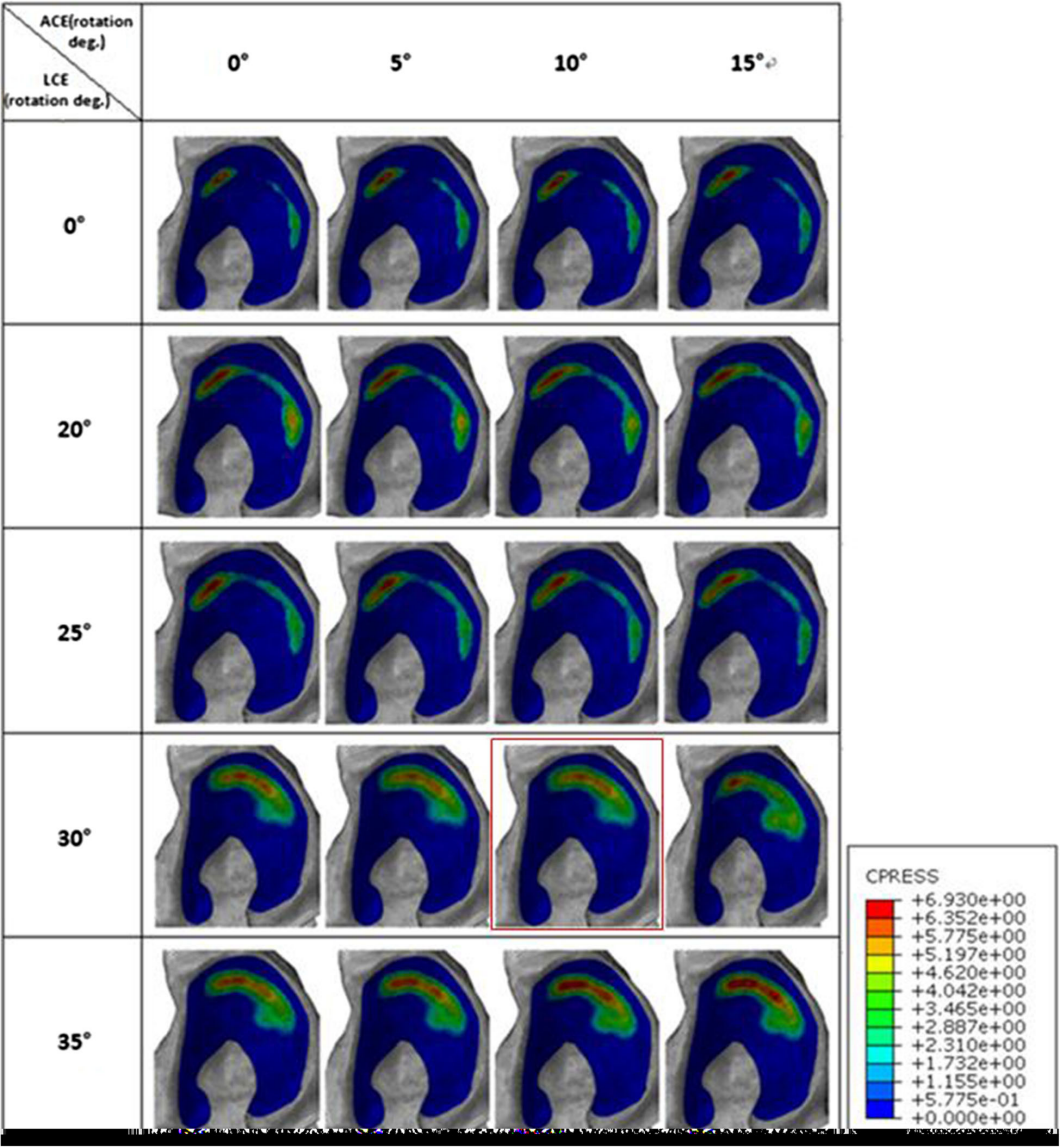
Contact pressure profile in acetabular cartilage of patient 3 at different CE angles. The *red box* means the optimal correction angle of patient 3, where the contact area was maximized and contact pressure and von Mises stress were minimized

The contact pressure profile in acetabular cartilage further confirmed that the contact area and pressure improved gradually as the acetabular fragment was corrected anterolaterally within a definite scope. However, the contact area did not improve significantly and even decreased sometimes, and the contact pressure rose gradually when the fragment was furtherly rotated outside of the scope. Figure 5 shows how the contact area and pressure changed in the acetabular cartilage as the acetabular fragment rotated anterolaterally in patient 3. As shown in the contact pressure profile in acetabular cartilage, the stress distribution in acetabular cartilage dispersed, and the peak contact pressure decreased; meanwhile, the stress concentration shifted from the edge of the superolateral region toward the medial of the superior region as the acetabular angle was corrected. The optimal position was reached when the acetabular fragment was rotated 30° laterally following a further 10° anterior rotation.

## Discussion

Because of its advantages such as the maintenance of an intact dorsal pillar without compromising the dimensions of the birth canal and allowing acetabulum reorientation in any directions [1], PAO has become the preferred choice as hip-preserving surgery for treating DDH patients. The critical and difficult part of the procedure is to confirm the optimal position of the acetabular fragment. There are many studies about FEA of PAO in DDH hips [19, 21, 27, 28]. However, the correlation between the optimal position and the severity of dysplasia was seldom investigated in these studies.

In this study, we performed FEA of PAO for three DDH patients with different severities of dysplasia, and we confirmed that the optimal position of the acetabular fragment is severity dependent and was not always corrected to the so-called radiological “normal” range. For example, patient 3 exhibited severe acetabular dysplasia with an original LCEA of −7° and an ACEA of 11°. The optimal position was when the fragment was rotated 30° laterally following further 10° anterior rotation, where the final LCEA of 25° and ACEA of 40° were both lower than the “normal” range. Patient 2 was a moderate acetabular dysplasia with an original LCEA of 7° and an ACEA of 22°, the optimal position was when the fragment was rotated 25° laterally following further 5° anterior rotation, where the final LCEA of 31° and ACEA of 51° were both within the “normal” range. Patient 1’s right hip exhibited a mild acetabular dysplasia with an original LCEA of 19° and an ACEA of 47°, the optimal position was when the fragment rotated 17° laterally, where the final LCEA of 36° and ACEA of 58° were slightly larger than the “normal” range.

The causes of these findings were analyzed. First, because of a serious deficiency of periacetabular bone stock for severe acetabular dysplasia coupled with the wavy morphology of acetabular rim, if we struggling to restore the acetabular angle within the so-called “normal” range for this severe dysplastic hip, a large rotation of the osteotomized fragment would be needed, therefore, the tension in soft tissue around the hip joint would increase accordingly, while the actual contact area of the articular cartilage would not increase significantly. Second, the one dimensional coverage deficiency was corrected, which could compromise the other dimensional coverage in three-dimensional space [29–31]. That is, the anterolateral coverage deficiency was corrected as the acetabular fragment was rotated, the posterior coverage deficiency was simultaneously aggravated, which would lead to hip joint instability. Third, femoroacetabular impingement maybe another adverse impact of a large rotation of the osteotomized fragment in three-dimensional space, which would shorten the distance between the acetabulum and the proximal femur. Ziebarth et al. [32] reported a high rate of femoroacetabular impingement after PAO, despite restoring the normal acetabular coverage. All of these causes will lead to disequilibrated mechanical transmission into the hip joint and finally cause the average contact pressure and von Mises stress increase in the articular cartilage. For mild and moderate DDH patients, due to the better development and congruence of the hip, the geometrical morphology was not altered significantly when the acetabulum was corrected to a slightly larger acetabular angle, and because of the increase of the acetabular coverage, the biomechanical environment of the hip can obtain the maximum level of improvement.

DDH is a complex musculoskeletal malformation, and PAO surgery can improve the contact area between acetabular cartilage and rebuild the near-normal biomechanical environment of the hip through the correction of the acetabular fragment, however, it cannot recover the normal anatomic structure of the hip. The parameter predictions of severe and moderate DDH (such as patients 2 and 3) are worse than those of normal hips. This may be the mechanical reason for poor prognosis of PAO for these severe and moderate DDH patients.

Finite element results also show that the contact area, average contact stress and von Mises stress did not significantly improve and even worsened when the acetabular fragment was rotated laterally following additional anterior rotation. Since the anterior coverage of the acetabulum increased simultaneously when the acetabular fragment was rotated laterally in three-dimensional space, a great anterior rotation would increase the risk of femoroacetabular impingement. Surgeons should therefore be careful with the anterior rotation of the acetabular fragment when performing PAO, especially for those DDH patients whose anterior coverage deficiency was not serious (such as patients 2 and 3).

This study has several limitations. First, as a numerical simulation investigation, FEA has its inherent limitations such as simplified treatment in the material properties and loading and boundary conditions, which may have some impact on the analytic results. Using similar experimental conditions, the numerical predictions were successfully corroborated against the results from a cadaveric experiment [25], which validated the FE model for this study. Second, only the loading condition of a single-legged stance was investigated in this study. Therefore, the effect of PAO on stress distribution in articular cartilage in loading conditions of other daily activities and other phase of the entire gait cycle could not be evaluated, and this is the focus of our future research. Third, only three subjects were studied; the sample size in this study was relatively small. Further investigations would include more subjects with different severities of dysplasia. Fourth, we did not consider the proximal femoral deformities in the analysis in this study. It is true that not only the correction of acetabular fragment but also the proximal femur influences the mechanical transmission in the hip joint. However, there are only a few DDH patients accompanied by proximal femoral deformities, especially for those mild and moderate DDH. Therefore, we focused on the effect of different severities of acetabular dysplasia on mechanical transmission in the hip joint in this study.

## Conclusions

In summary, our FE models demonstrate the clinical hypothesis from the view of mechanics that the optimal corrective position of the acetabular fragment is severity dependent rather than within the radiological “normal” range for all DDH patients. We prudently propose that the optimal correction CE angle of mild, moderate, and severe DDH is slightly larger than the “normal” range, within the “normal” range, and less than the lower limit of the “normal” range, respectively, and hope that this information could help orthopedic surgeons in customized surgical planning.

## Additional files

Additional file 1: Figure S1. The STL model of the hip generated by Mimics 10.01. (TIF 1 MB)
Additional file 2: Figure S2. The 3D surface model of the hip generated by Geomagic 12.0. (TIF 1 MB)
Additional file 3: Supplementary table. The contact area, verage contact pressure and Von Mises stress in the acetabular cartilage of patient 1's right hip, patient 2 and patient 3 at different CE angles. (DOCX 19 kb)

## Abbreviations

ACEA: Anterior center-edge angle
DDH: Developmental dysplasia of the hip
FEA: Finite element analysis
LCEA: Lateral center-edge angle
PAO: Bernese periacetabular osteotomy
