# Supplementary figures and images for: Does the optimal position of the acetabular fragment should be within the radiological normal range for all developmental dysplasia of the hip? A patient-specific finite element analysis
Source: J Orthop Surg Res. 2016 Oct 4;11:109. doi: 10.1186/s13018-016-0445-3 (PMC5050724; doi:10.1186/s13018-016-0445-3)

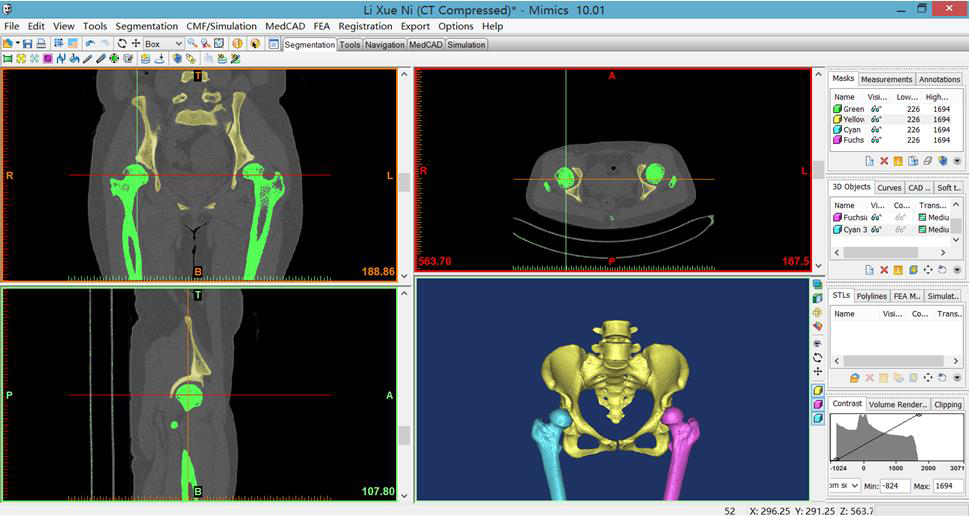

Supplement: Additional file 1: Figure S1. — The STL model of the hip generated by Mimics 10.01. (TIF 1 MB) [file 13018_2016_445_MOESM1_ESM.tif]

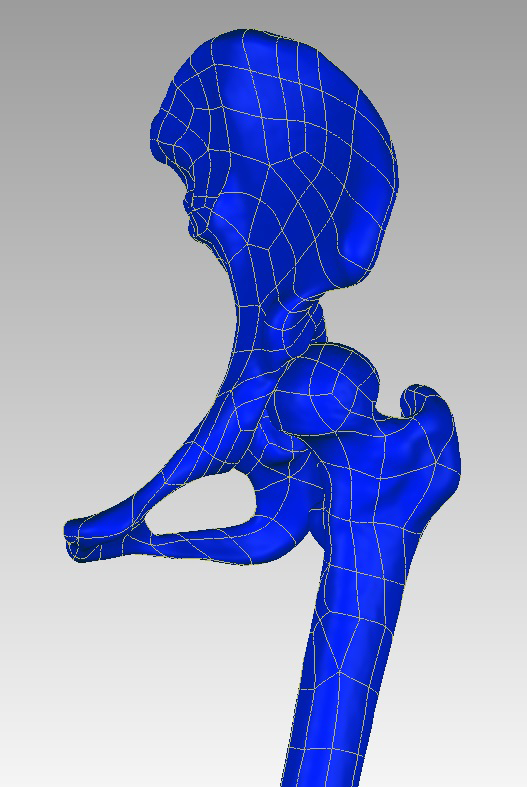

Supplement: Additional file 2: Figure S2. — The 3D surface model of the hip generated by Geomagic 12.0. (TIF 1 MB) [file 13018_2016_445_MOESM2_ESM.tif]
