# Supplementary material for: Does the optimal position of the acetabular fragment should be within the radiological normal range for all developmental dysplasia of the hip? A patient-specific finite element analysis
Source: J Orthop Surg Res. 2016 Oct 4;11:109. doi: 10.1186/s13018-016-0445-3 (PMC5050724; doi:10.1186/s13018-016-0445-3)
Supplement: Additional file 3: — Supplementary table. The contact area, verage contact pressure and Von Mises stress in the acetabular cartilage of patient 1's right hip, patient 2 and patient 3 at different CE angles. (DOCX 19 kb) [file 13018_2016_445_MOESM3_ESM.docx]

**Table S1** **Contact area in the acetabular cartilage of patient 1's right hip at different CE angles(mm²)**

| Lateral  rotation (°) | Anterior rotation (°) | | | |
| --- | --- | --- | --- | --- |
|  | 0 | 5 | 10 | 15 |
| 0 | 327.067 | 233.632 | 230.535 | 232.754 |
| 10 | 336.276 | 338.530 | 339.157 | 342.673 |
| 15 | 351.639 | 349.567 | 350.173 | 352.037 |
| 17 | 388.472 | 389.783 | 388.076 | 376.900 |
| 20 | 373.650 | 370.492 | 374.005 | 369.643 |

The red box indicate the maximum contact area.

**Table S2 Contact area in the acetabular cartilage of patient 2 at different CE angles(mm²)**

| Lateral  rotation (°) | Anterior rotation (°) | | | |
| --- | --- | --- | --- | --- |
|  | 0 | 5 | 10 | 15 |
| 0 | 276.247 | 278.573 | 283.953 | 290.459 |
| 15 | 306.186 | 309.473 | 319.199 | 319.918 |
| 20 | 335.093 | 349.682 | 350.742 | 350.694 |
| 25 | 368.368 | 370.520 | 370.523 | 368.406 |
| 30 | 360.069 | 365.660 | 366.332 | 353.798 |

The red box indicate the maximum contact area.

**Table S3 Contact area in the acetabular cartilage of patient 3 at different CE angles(mm²)**

| Lateral  rotation (°) | Anterior rotation (°) | | | |
| --- | --- | --- | --- | --- |
|  | 0 | 5 | 10 | 15 |
| 0 | 225.634 | 229.749 | 235.952 | 240.68 |
| 20 | 296.816 | 297.035 | 303.666 | 305.948 |
| 25 | 306.186 | 309.473 | 319.199 | 319.918 |
| 30 | 349.024 | 349.784 | 354.674 | 348.357 |
| 35 | 351.567 | 351.880 | 351.757 | 352.206 |

The red box indicate the maximum contact area.

**Table S4 Average contact pressure in the acetabular cartilage of patient 1's right hip at different CE angles(MPa)**

| Lateral  rotation (°) | Anterior rotation (°) | | | |
| --- | --- | --- | --- | --- |
|  | 0 | 5 | 10 | 15 |
| 0 | 5.759 | 5.604 | 5.743 | 5.787 |
| 10 | 5.307 | 5.253 | 5.318 | 5.355 |
| 15 | 4.965 | 4.762 | 4.975 | 5.005 |
| 17 | 4.604 | 4.617 | 4.609 | 4.621 |
| 20 | 4.726 | 4.730 | 4.722 | 4.727 |

The red box indicate the minimum contact pressure.

**Table S5 Average contact pressure in the acetabular cartilage of patient 2 at different CE angles(MPa)**

| Lateral  rotation (°) | Anterior rotation (°) | | | |
| --- | --- | --- | --- | --- |
|  | 0 | 5 | 10 | 15 |
| 0 | 6.328 | 6.316 | 6.307 | 6.219 |
| 15 | 5.614 | 5.506 | 5.331 | 5.243 |
| 20 | 5.203 | 5.189 | 4.875 | 5.067 |
| 25 | 4.716 | 4.694 | 4.708 | 4.730 |
| 30 | 4.712 | 4.713 | 4.814 | 4.819 |

The red box indicate the minimum contact pressure.

**Table S6 Average contact pressure in the acetabular cartilage of patient 3 at different CE angles(MPa)**

| Lateral  rotation (°) | Anterior rotation (°) | | | |
| --- | --- | --- | --- | --- |
|  | 0 | 5 | 10 | 15 |
| 0 | 6.935 | 6.886 | 6.763 | 6.430 |
| 20 | 6.283 | 6.247 | 6.195 | 6.161 |
| 25 | 6.150 | 5.838 | 5.764 | 5.608 |
| 30 | 5.337 | 5.218 | 5.003 | 5.456 |
| 35 | 5.497 | 5.474 | 5.506 | 5.568 |

The red box indicate the minimum contact pressure.

**Table S7. Von Mises stress in the acetabular cartilage of patient 1's right hip at different CE angles(MPa)**

| Lateral  rotation (°) | Anterior rotation (°) | | | |
| --- | --- | --- | --- | --- |
|  | 0 | 5 | 10 | 15 |
| 0 | 2.169 | 2.147 | 2.160 | 2.171 |
| 10 | 1.885 | 1.840 | 1.846 | 1.895 |
| 15 | 1.766 | 1.746 | 1.769 | 1.774 |
| 17 | 1.607 | 1.614 | 1.618 | 1.681 |
| 20 | 1.638 | 1.641 | 1.686 | 1.707 |

The red box indicate the minimum Von Mises stress.

**Table S8. Von Mises stress in the acetabular cartilage of patient 2 at different CE angles(MPa)**

| Lateral  rotation (°) | Anterior rotation (°) | | | |
| --- | --- | --- | --- | --- |
|  | 0 | 5 | 10 | 15 |
| 0 | 2.393 | 2.504 | 2.437 | 2.428 |
| 15 | 2.107 | 2.017 | 1.916 | 1.912 |
| 20 | 1.898 | 1.816 | 1.867 | 1.903 |
| 25 | 1.725 | 1.643 | 1.649 | 1.739 |
| 30 | 1.724 | 1.721 | 1.755 | 1.761 |

The red box indicate the minimum Von Mises stress.

**Table S9. Von Mises stress in the acetabular cartilage of patient 3 at different CE angles(MPa)**

| Lateral  rotation (°) | Anterior rotation (°) | | | |
| --- | --- | --- | --- | --- |
|  | 0 | 5 | 10 | 15 |
| 0 | 2.514 | 2.484 | 2.435 | 2.351 |
| 20 | 2.279 | 2.246 | 2.232 | 2.227 |
| 25 | 2.218 | 2.116 | 2.097 | 2.080 |
| 30 | 1.858 | 1.833 | 1.714 | 1.893 |
| 35 | 1.942 | 1.973 | 2.009 | 2.028 |

The red box indicate the minimum Von Mises stress.
